# Brine-Processed *Caulerpa lentillifera* Macroalgal Stability: Physicochemical, Nutritional and Microbiological Properties

**DOI:** 10.3390/life13112112

**Published:** 2023-10-25

**Authors:** Wanida Pan-utai, Prajongwate Satmalee, Safiah Saah, Yupadee Paopun, Montakan Tamtin

**Affiliations:** 1 Department of Applied Microbiology, Institute of Food Research and Product Development, Kasetsart University, Bangkok 10900, Thailand; 2 Department of Food Chemistry and Physics, Institute of Food Research and Product Development, Kasetsart University, Bangkok 10900, Thailand; ifrpws@ku.ac.th; 3 Department of Nutrition and Health, Institute of Food Research and Product Development, Kasetsart University, Bangkok 10900, Thailand; safiah.s@ku.th; 4 Scientific Equipment and Research Division, Kasetsart University Research and Development Institute, Kasetsart University, Bangkok 10900, Thailand; rdiydp@ku.ac.th; 5 Department of Fisheries, Ministry of Agriculture and Cooperatives, Kung Krabaen Bay Royal Development Study Center, Chantha Buri 22120, Thailand; mtamtin@hotmail.com

**Keywords:** macroalgae, *Caulerpa*, brine, salt, storage, properties

## Abstract

*Caulerpa lentillifera* is a type of green macroalga that is commonly consumed as fresh seaweed, particularly in Southeast Asia. The effects of different salt types and concentrations on C. lentillifera during brine processing were investigated using table, sea and flower salt at 10–30% levels. The colour and texture of *C. lentillifera* varied across different treatments. After storage in brine for 12 weeks, lightness (L*) decreased, greenness (a*) decreased and yellowness (b*) increased while firmness increased in all treatments compared to fresh algae. The nutritional composition did not change significantly over time. To ensure the safety and quality of seaweed for consumption, the optimal salt level for brine processing should not exceed 30% table salt. The morphology and elements contained in different types of salt were also observed, and the microbiological safety of seaweed was evaluated. The popularity of *Caulerpa* macroalgae is rapidly increasing among consumers, leading to a growing demand for ready-to-eat *Caulerpa* products. However, food safety and security standards must be maintained.

## 1. Introduction

*Caulerpa lentillifera*, a green macroalga termed edible sea grapes, is widely consumed as fresh seaweed in Southeast Asia [1]. This plant-based food fulfils a niche market and shows potential for expansion as a sustainable resource [2,3], with many seaweed products now produced and distributed for human consumption [4]. *C. lentillifera*, called ‘green caviar’ in Europe, ‘umibudo’ in Japan and ‘rong nho’ in Vietnam, is in high demand, particularly in Southeast Asia. This siphonous alga has a unique texture consisting of nutritional, edible fronds bearing vesiculate ramuli and horizontal stolons with rhizomes [5]. Market demand for seaweed has recently increased. Globally, seaweed farming output has increased 1000-fold from 1950 to 2019 [6], while green seaweed output in 2019 was 16,696 tonnes, accounting for only 0.05% of the annual overall seaweed production. Green seaweeds such as *Caulerpa* sp., *Ulva prolifera*, *Monostroma nitidum*, *Capsosiphon fulvescens* and *Codium fragile* comprised part of the 2019 output, and these species are included in the FAO’s Aquatic Sciences and Fisheries Information System. Only 7 of the 100 known *Caulerpa* species are used for human consumption, with *C. lentillifera* and *C. racemosa* being the leading varieties [7].

*Caulerpa* alga is rich in proteins, carbohydrates, lipids and pigments, with promising biological features [8,9]. *C. lentillifera* contains high concentrations of polyunsaturated fatty acids, minerals, vitamins and bioactive substances that provide several health advantages. However, few articles have addressed its nutritional content and possible health advantages [1]. Natural metabolites are now understood to be crucial elements of human meals due to their ability to combat metabolic syndrome and vitamin deficiency [10], with both primary and secondary metabolites having varied physiological effects on health and illness. These metabolites satisfy numerous biological and functional requirements and show antiproliferative, immunostimulatory, antioxidant, antibacterial and anticancer capabilities [11,12,13].

*C. lentillifera* is becoming increasingly popular as a sea vegetable used for human food [2]. Seaweeds have a high moisture content and nutritional richness, but this encourages microbial development, leading to a limited shelf life [14]. Traditional techniques for preserving seaweed include drying and salting, which are both based on decreasing water activity (a_w_). High levels of chemical and microbiological stability are achieved via drying; however, unwanted microbial contaminants, which may include pathogenic species, are not entirely removed [15]. Rehydration of *Caulerpa* after drying does not produce a product with fresh seaweed characteristics, while after drying, the final product may not be suitable for various purposes [16]. Salting is another effective method for preserving high-moisture foods and can be used as a stand-alone operation or for drying vegetables and other foods [17]. High salinity is an efficient method for preserving seaweed, but salted seaweed can still degrade during long-term storage [18]. Brine solutions protect the microbiological quality of fresh foods by lowering water activity, inducing hyperosmotic shock and altering the metabolism of spoilage and harmful microbes via direct lethal actions of the chloride ion [19,20]. Brine seaweeds are used alongside fresh ingredients in various cuisines after washing in fresh water. Many businesses are interested in producing salted kelp products to satisfy regional niche markets by promoting organic, raw or minimally processed consumer trends. However, science-based evidence on the optimal processing procedures to provide safe, high-quality goods with a long shelf life is lacking [16]. *C. lentillifera* fresh seaweed fills a niche market in Thailand, and production is supported by the Ministry of Agriculture and Cooperatives. The productivity of *C. lentillifera* depends on several factors, while the environment during seaweed production in large open systems is largely uncontrolled. Production during optimal climate conditions produces more biomass. The limited storage time of *C. lentillifera* during periods of oversupply is also a bottleneck in satisfying the continuous demand for fresh seaweed. Salt is one of the best methods to ensure food safety and preservation due to its ability to decrease the water activity in food [21]. Traditionally, salt has been used in food processing to extend the shelf life of products during times of scarcity [22], which several different kinds of salt being utilised. Sodium chloride, also known as table salt, is commonly added to processed foods to preserve the flavour and texture [23]. Recently, natural sea salts have increased in popularity. Sea salt is produced by evaporating seawater in shallow pools called salterns and then harvesting and packing the salt for use as a food ingredient [24]. Different elements in salts impact food processing. The effects of sodium chloride and calcium chloride salt types on the growth of lactic acid bacteria and pathogens during vegetable fermentation were studied [25]. Controversy surrounds the mechanism through which calcium inhibits the softening of vegetable tissue [26]. Optimal fruit colour was obtained at an equal ratio between potassium chloride and sodium chloride or calcium chloride [27]. However, various kinds of salts contain different elements that diversely affect seaweed quality under brine processing. Therefore, this study investigated how brine preservative methods impacted the quality of *C. lentillifera* during storage. Physicochemical, nutritional and microbiological properties were observed to improve the stability of ready-to-eat *C. lentillifera*, with its recovery from rehydration being similar to that observed in the original product.

## 2. Materials and Methods

### 2.1. Materials

Sea grapes, *C. lentillifera,* were purchased from a commercial organic farm (Family Farm) in Phetchaburi Province, Thailand. The *C. lentillifera* strain was distributed by the Coastal Aquaculture Research and Development Division, Department of Fisheries, Ministry of Agriculture and Cooperatives, Thailand. Sea Grapes Farm cultured and harvested the fresh macroalgae for premium food-grade production following the Standard Farm Regulations announced by the Ministry of Agriculture and Cooperatives. All experiments used fresh biomass of *C. lentillifera* as the raw material for seaweed brine salt. Three kinds of salts were used in the experiments, including commercial table salt for cooking (Prung Thip, Thai Refined Salt Co., Ltd., Bangkok, Thailand), sea salt and flower salt from a local salt farm (Local Salt Farm, Samut Sakhon, Khok Kham, Thailand), Thailand (13°37′04.5″ N 100°15′36.0″ E).

### 2.2. Brine Salt Experiments

Fresh *C. lentillifera* was brined using the three types of salt under concentrations of 10, 20 and 30% (*w*/*v*). Fresh *C. lentillifera* (500 g wet weight) was immersed in 1 L of salt solution, packed in clear polypropylene bags of 120-micron thickness and vacuum-sealed using a sealer machine. The brine salt samples were stored at 25 °C in the dark. All experiments were performed in triplicate.

### 2.3. Analysis Methods

Fresh *C. lentillifera* and brine salt samples were evaluated for physicochemical, nutritional and microbiological properties. Biomass of *C. lentillifera* brine samples was prepared for rehydration before analysis. Brine salt samples were drained, followed by washing off the residue salt solution with distilled water at a ratio of 1:20 *w*/*v* five times. Each cycle set lasted 10 min. The samples were then drained, and the final distilled residue was used for further analysis.

#### 2.3.1. Physicochemical Analysis

##### Colour Measurement

The colour of fresh and *C. lentillifera* brine samples was determined using a Datacolour Spectraflash Spectrophotometer (SF 600 plus, Datacolour International Co., Lawrenceville, NJ, USA). The colour was expressed in terms of lightness L*, from black (0) to white (100), with chromaticity parameters a* ranging from green (−) to red (+) and b* from blue (−) to yellow (+).

##### Texture Analysis

The hardness and firmness of fresh *C. lentillifera* and brine samples were determined using a Texture Analyzer (TA-XTplus, Stable Micro Systems, Godalming, UK) with a 35 mm diameter cylinder probe (P35) and crisp fracture support rig. A feed rate of 2 mm/s and 70% stress–strain for 5 s controlled the speed. Hardness and firmness results were recorded as the maximum force (g) and calculated from the slope of the first peak (g/s).

#### 2.3.2. Nutritional Analysis

The nutritional compositions of fresh *C. lentillifera* and brine samples were analysed according to AOAC methods [28]. Moisture content was determined by oven-drying samples at 105 °C to constant weight. The ash content was examined by burning the dried samples at 550 °C in an electric furnace, while crude protein content was determined using the Kjeldahl method with a nitrogen conversion factor of 6.25. Crude fat content was evaluated using the modified Bligh and Dyer technique [29]. The samples were resuspended in a reagent containing distilled water, methanol and chloroform at ratios of 0.8:2.0:1.0 and well mixed. The mixture was then placed in an ultrasonic bath for 15 min before centrifugation at 3461× *g* for 15 min to separate the lipids. The lipid phase was collected, and the reagent was mixed with the cell residual debris for several extractions until the cells were colourless. The lipid extract was then filtered from the contaminated cell debris using Whatman filter paper GF/C and dried at 80 °C to constant weight [30]. The amount of crude fibre was measured using acid and alkaline digestion, and the extracted fibre was dried to constant weight. Differences in results were utilised to compute carbohydrate, total ash, crude protein, crude fat and ash contents in 100 g of dry matter.

#### 2.3.3. Microbiological Analysis

Aerobic plate counts of yeast, mould and coliform bacteria of fresh *C. lentillifera* and brine samples were determined using FDA standard methods. The aerobic plate count (APC) was counted on plate count agar (PCA) incubated at 35 °C for 48 h [31]. Yeast and mould countable samples were determined using Dichloran 18% glycerol (DG18) agar and incubated at 25 °C [32]. Coliform bacteria were analysed using Chromocult agar and incubated at 35 °C for 24 h [33].

### 2.4. Morphological and Elemental Analysis

The morphology and various elements or ions contained in the salt formulations were related to the salt solubility, mineralogy, salinity and conductivity of the solutions [34]. The three different kinds of salt were evaluated via morphological analysis using a scanning electron microscope and energy-dispersive X-ray spectrometer (SEM-EDS, model JSM-5410LV and EDS, element and chemical analysis via Oxford Energy-Dispersive X-ray, Hitachi High-Technologies Corporation, Tokyo, Japan). The salt samples were carefully placed in a pressure-controlled analysis chamber and connected to the EDS sample holder. Samples were not carbon-coated for EDS to determine the actual elemental composition. After 10 to 15 min, the gadget calculated the weights (%) and atomic weights (%) of the sample’s constituent elements. The EDS analyser determined the total component atomic weight of each element in the salt samples.

### 2.5. Statistical Analysis

All experiments were analysed in triplicate, with all parameters statistically analysed via one-way analysis of variance (ANOVA) using SPP 12.0 (SPSS, Inc., Chicago, IL, USA). Multiple comparisons in each group were conducted using Duncan’s Multiple Range Test (DMRT) with significance set at 0.05 (*p* < 0.05).

## 3. Results

*C. lentillifera* macroalgae were stored under different kinds and concentrations of salt solutions to assess improvements in the stability of algal plant-based food. All experiments displayed the flabby appearance of seaweed and quick recovery after rehydration. The physicochemical, nutritional and microbiological properties were evaluated during storage for 12 weeks, while the morphological and elemental compositions of the salts used as the preservative ingredients were also investigated.

### 3.1. Physicochemical Characterization of C. lentillifera

Physicochemical properties are critical characteristics of fresh edible seaweed, with colour affecting acceptance. The colour parameters of various combined *C. lentillifera* macroalgal conditions are shown in Table 1, Table 2 and Table 3 for table, sea and flower salt, respectively. Fresh *C. lentillifera* macroalga, as the initial sample, had lightness (L*), greenness (a*) and yellowness (b*) values of 29.92, −1.01 and 2.87, respectively. After 12 weeks of storage in brine, *C. lentillifera* in all experiments presented decreased lightness (L*), decreased greenness (a*) and increased yellowness (b*). The colour values of *C. lentillifera* samples stored under 10–30% table salt are shown in Table 1. Colour parameter changes showed the same tendency for different salt concentrations during the 12 weeks of storage. L* values decreased to 19.75–21.52, with a* values ranging from 1.48 to 1.56 and b* values from 5.87 to 6.41 after 12 weeks of storage. The colour parameters of *C. lentillifera* under 10–30% sea salt concentrations are shown in Table 2. The L* and a* values decreased, whereas the b* values increased after 12 weeks of storage, ranging from 22.41 to 22.75, 0.50 to 0.62 and 5.26 to 5.64, respectively. At a higher content of sea salt, the remaining green colour changed over the storage duration. Table 3 shows *C. lentillifera* colour parameters under 10–30% flower salt concentrations. L* decreased to around 21 after 12 weeks of storage with a* decreasing and b* increasing, ranging from 1.33 to 1.55 and 4.14 to 6.40, respectively. The highest flower salt concentration, 30%, showed a slow decrease in a* over the storage duration. The *C. lentillifera* samples showed changes in colour during storage and were non-stable compared with the fresh sample. However, other parameters remained a concern during storage.

The textural properties of *C. lentillifera* under brine conditions, expressed as hardness and firmness from rehydration, are shown in Table 4, Table 5 and Table 6. Fresh *C. lentillifera* gave hardness and firmness values of 1150 g force and 423 g/s, respectively. All experimental results show increased hardness and firmness of *C. lentillifera* after 12 weeks of brine storage. Higher concentrations of each kind of salt gave increased hardness and firmness parameters. Table 4 shows the hardness and firmness of *C. lentillifera* stored under table salt for 12 weeks. The textural results of *C. lentillifera* stored in sea and flower salt showed similar tendencies (Table 5 and Table 6). Higher salt concentrations directly affected the hardness and firmness of seaweed stability. The colour and texture of *C. lentillifera* were acceptable as seen with the naked eye with a good overall appearance.

### 3.2. Nutritional Composition of C. lentillifera Stability

Algae or seaweed are rich sources of nutrients as a sustainable food supply for the future [35]. The nutritional compositions of fresh seaweed and *C. lentillifera* stored under table salt are shown in Table 7. *C. lentillifera* brined in sea salt and flower salt had a poor nutritional composition. High moisture contents of fresh and brine-stored *C. lentillifera* were obtained at 95–98% due to the high water content in the samples. Ash content had the highest value in fresh seaweed. Fresh *C. lentillifera* recorded ash, fat, protein, fibre and carbohydrate contents of 65.6 6.3, 4.9, 4.1 and 19.1% dry weight (DW), respectively. After 12 weeks of brine storage, *C. lentillifera* was mainly composed of carbohydrates, whereas ash content decreased. Overall nutritional compositions did not differ between storage durations, while the nutritional value of sea grapes depended on the original seaweed material used.

### 3.3. Food Safety

Microbiological determinations of fresh and brine-stored *C. lentillifera* were evaluated for food safety at various conditions, with results shown in Table 8. Fresh *C. lentillifera* was evaluated for the aerobic plate count, yeast, mould, and coliform were 1.40 × 10^4^ CFU/g, <10 CFU/g, 2.30 × 10^2^ CFU/g, and 9.87 MPN/g, respectively. During storage, total plate count increased as salt concentration increased, indicating microbial contamination in salt ingredients, especially in sea and flower salts. Coliform bacteria were found in the initial seaweed showing contamination from flower salt as the source of preservative. Thus, salt ingredients were affected by the physic, proximate and microbial contaminated properties, which assisted in extending the stability and preservation of the seaweed products during the 12 weeks of storage.

## 4. Discussion

*C. lentillifera*, also known as sea grapes, is a seaweed commonly consumed as a fresh vegetable, particularly in Southeast Asia [1]. Interest in this food product is increasing in Europe as the demand for vegetarian/vegan food items and knowledge of health and environmental problems associated with food choices increases [36]. *C. lentillifera* has a distinct texture and good nutritional components for human nutrition [37]. Fresh sea grapes have been marketed to domestic and foreign markets due to their high nutritional value [38]. After harvest, fresh *C. lentillifera* is still alive and actively photosynthesising, with post-harvest management a delicate stage of the life cycle. The shelf life of the product is short requiring swift retail and transportation [7]. Improvement of product shelf life would be beneficial as an alternative ingredient for food courses. Copious research has investigated the properties of fresh *C. lentillifera* during storage.

*Caulerpa* sp. is an edible green macroalgae that contains bioactive compounds [39]. The colour and texture of brine-soaked *C. lentillifera* are similar to those of the fresh product and attractive to customers. Green algae contain chlorophyll as an excellent natural colourant with antioxidant properties including chlorophyll a (26.82%) and chlorophyll b (12.91%) [40]. Fresh *C. lentillifera* appears green to the naked eye. The green alga *Caulerpa* is characterised by a green colour imparted by a high concentration of bioactive pigments such as chlorophylls. *Caulerpa* sp. contains carotenoids including the xanthophylls carotene, lutein, astaxanthin, canthaxanthin, zeaxanthin and fucoxanthin [41]. Our results show that brine-stored *C. lentillifera* under different salt types and concentrations showed decreasing colour and textural parameters. A reduction in the green colour occurs because the b* colour value becomes more intense as the chlorophyll breaks down [42], while *C. lentillifera* quality is acceptable after recovery through rehydrationFurthermore, the increased salt concentration may cause osmotic equalises to the salt solution within and outside the cell membrane, leading to increased hardness and firmness after rehydration in *C. lentillifera.*

*C. lentillifera* is a consumable seaweed with a high moisture content and nutritional richness that encourages microbial development [15]. Conventional processes utilized to preserve seaweed and extend product shelf life include drying, freezing and salting. However, neither drying nor freezing can preserve seaweeds in their original form [43], while rehydration does not reproduce product quality. Salting is an alternative preservation process that has been reported for *Halorubrum laminariae* and other seaweeds [44]. Our results show that the physicochemical properties of *C. lentillifera* stored in brine changed during storage. Seaweed must be desalted in water before ingestion, which may result in the loss of nutrients and phytochemical components [15]. The nutritional contents of *C. lentillifera* depend on several factors during cultivation, including irradiance and temperature [5]. Similar reports from different countries support a wide variety of proximate compositions [1,45] Our results for *C. lentillifera* stored in brine showed various biochemical changes over time, concurring with a previous report in which *C. lentillifera* nutritional characterisation changed during dehydration, leading to oxidative stress [46]. Microorganisms also pose a problem for food safety. Microbiological analysis of the table salt condition showed the least number of microbes, whereas the flower salt condition had the highest microbial contamination.

**Figure 1:**
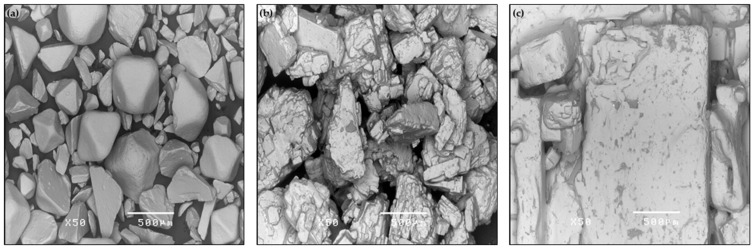
Structure of different kinds of salts under scanning electron microscopy with magnification 50×: (**a**) table (**b**) sea and (**c**) flower salts.

The evaluation of the salts’ morphology indicated that the salt solution’s solubility was related to the processing time. In contrast, the ion composition of salt was observed in the salt solution and sea grapes after rehydration remained. Elemental and morphological salt ingredients of the brine *C. lentillifera* process were determined, as shown in Table 9 and Figure 1, respectively. The results show a variety of elements in sea salt, whereas table salt mainly contained oxygen, sodium and chloride ions. However, table salt compositions were obtained the similar re Sea and flower salts contained other elements including magnesium, sulphur and potassium. Calcium was only found in flower salt. However, the previous results showed that multiple elements were obtained from South Korean, Chinese, and Greek salts [47,48]. Therefore, the variety of elements in salt depends on the location of salt production. Different kinds of salts used for the brining process impacted ion accumulation in *C. lentillifera* during storage. Due to the concentration difference, water transfers from foods to brine while salt transfers from brine to foods due to osmosis while brining. Brine has a larger ion concentration than fibre cells, and salt ions diffuse through cell membranes until they achieve equilibrium [49]. The structure of the different kinds of salts was observed under a scanning electron microscope (SEM). Table salt had the smallest grain size and the highest solubility, in the order table > sea > flower salt, while salt concentration and ion content affected membrane breakdown during the brining process. All the parameters indicated that table salt was the most suitable for the brining process of *C. lentillifera* for human consumption and safety during storage. Sea and flower salts contain numerous interesting ions. Sterilisation of salt solutions is a safe option to eliminate pathogens before the brining process. The food industry and health authorities use microbiology tests to confirm food safety at the microbiological level to enhance decision -making and safety across the food chain [50]. Particular spoilage bacteria also multiply and reduce fresh product shelf life [51].

## 5. Conclusions

The macroalga *C. lentillifera* is a significant source of marine food and is commercially produced in large quantities, especially Southeast Asia. However, certain seasons are not suitable for widespread commercial manufacture. Fresh *C. lentillifera* has a short shelf life, and brining is used to extend its storage duration. Various types and concentrations of salts were studied. In all pickling experiments, product hardness increased compared to fresh algae. Pickling increases the shelf life of fresh *Cauperpa* macroalgae. To ensure high quality and food safety during seaweed storage, the optimal salt concentration for brine processing should not exceed 30% table salt. Sea and flower salt can also be used as potential preservatives, but the product requires sterilisation before use. *Caulerpa* macroalgae is globally accepted by consumers, and the demand for ready-to-eat *Caulerpa* products, previously a specialized market, is rapidly increasing. However, food safety and security aspects must also be continuously monitored.

## 6. Patents

The preparation methods of sea grapes and brine salt formulations in this research study are covered under a Thailand petty patent.

## Figures and Tables

**Table 1:** Colour values of *C. lentillifera* under 10–30% table salt concentrations during 12 weeks of storage.

Weeks	Salt Concentration		
	10%	20%	30%
L*			
0	29.92 ^d^ ± 0.34	29.92 ^e^ ± 0.34	29.92 ^e^ ± 0.34
2	25.76 ^b^ ± 0.38	24.61 ^b^ ± 0.08	24.85 ^c^ ± 0.37
4	25.83 ^b^ ± 0.31	24.64 ^bc^ ± 0.11	24.46 ^b^ ± 0.13
6	26.33 ^c^ ± 0.18	25.13 ^d^ ± 0.39	25.39 ^d^ ± 0.29
8	25.74 ^b^ ± 0.36	24.98 ^cd^ ± 0.35	25.27 ^d^ ± 0.14
10	21.49 ^a^ ± 0.16	19.86 ^a^ ± 0.35	20.39 ^a^ ± 0.15
12	21.52 ^a^ ± 0.14	19.75 ^a^ ± 0.11	20.46 ^a^ ± 0.16
a*			
0	−1.01 ^a^ ± 0.18	−1.01 ^a^ ± 0.18	−1.01 ^a^ ± 0.18
2	0.72 ^d^ ± 0.04	0.67 ^d^ ± 0.05	0.81 ^d^ ± 0.01
4	0.54 ^c^ ± 0.04	0.13 ^b^ ± 0.02	−0.14 ^b^ ± 0.03
6	0.35 ^b^ ± 0.07	0.68 ^d^ ± 0.07	0.42 ^c^ ± 0.07
8	0.42 ^b^ ± 0.08	0.50 ^c^ ± 0.06	0.51 ^c^ ± 0.07
10	1.61 ^f^ ± 0.06	1.51 ^e^ ± 0.03	1.39 ^e^ ± 0.10
12	1.50 ^e^ ± 0.07	1.56 ^e^ ± 0.07	1.48 ^e^ ± 0.11
b*			
0	2.87 ^a^ ± 0.79	2.87 ^a^ ± 0.79	2.87 ^a^ ± 0.79
2	7.27 ^e^ ± 0.26	4.29 ^c^ ± 0.19	5.80 ^c^ ± 0.32
4	4.86 ^c^ ± 0.20	3.76 ^b^ ± 0.05	3.57 ^b^ ± 0.07
6	4.05 ^b^ ± 0.27	3.68 ^b^ ± 0.08	3.55 ^b^ ± 0.07
8	3.71 ^b^ ± 0.13	3.70 ^b^ ± 0.06	3.59 ^b^ ± 0.04
10	6.41 ^d^ ± 0.16	6.23 ^d^ ± 0.18	5.78 ^c^ ± 0.17
12	6.41 ^d^ ± 0.13	6.29 ^d^ ± 0.09	5.87 ^c^ ± 0.33

Data in the same parameter and column with different superscript letters are significantly different (*p* < 0.05).

**Table 2:** Colour values of *C. lentillifera* under 10–30% sea salt concentrations during 12 weeks of storage.

Weeks	Salt Concentration		
	10%	20%	30%
L*			
0	29.92 ^c^ ± 0.34	29.92 ^d^ ± 0.34	29.92 ^d^ ± 0.34
2	30.07 ^c^ ± 0.42	25.79 ^c^ ± 0.35	26.55 ^c^ ± 0.10
4	30.07 ^c^ ± 0.42	25.79 ^c^ ± 0.35	26.55 ^c^ ± 0.10
6	24.95 ^b^ ± 0.38	24.97 ^b^ ± 0.35	25.88 ^b^ ± 0.27
8	25.19 ^b^ ± 0.22	24.98 ^b^ ± 0.32	26.04 ^b^ ± 0.46
10	22.50 ^a^ ± 0.13	22.74 ^a^ ± 0.39	22.62 ^a^ ± 0.29
12	22.41 ^a^ ± 0.17	22.75 ^a^ ± 0.21	22.47 ^a^ ± 0.09
a*			
0	−1.01 ^a^ ± 0.18	−1.01 ^a^ ± 0.18	−1.01 ^a^ ± 0.18
2	0.92 ^d^ ± 0.13	−0.54 ^bc^ ± 0.08	−0.43 ^b^ ± 0.07
4	0.86 ^d^ ± 0.06	−0.58 ^b^ ± 0.07	−0.43 ^b^ ± 0.0
6	0.67 ^c^ ± 0.13	−0.43 ^c^ ± 0.13	−0.41 ^b^ ± 0.07
8	0.54 ^b^ ± 0.07	−0.30 ^d^ ± 0.08	−0.30 ^c^ ± 0.06
10	0.54 ^b^ ± 0.07	0.57 ^e^ ± 0.07	0.62 ^d^ ± 0.05
12	0.50 ^b^ ± 0.07	0.59 ^e^ ± 0.10	0.62 ^d^ ± 0.04
b*			
0	2.87 ^a^ ± 0.79	2.87 ^a^ ± 0.79	2.87 ^a^ ± 0.79
2	5.07 ^b^ ± 0.18	3.52 ^b^ ± 0.19	2.86 ^a^ ± 0.22
4	5.07 ^b^ ± 0.18	3.52 ^b^ ± 0.19	2.86 ^a^ ± 0.22
6	5.45 ^bc^ ± 0.13	3.92 ^c^ ± 0.20	3.22 ^a^ ± 0.32
8	5.25 ^b^ ± 0.09	4.12 ^c^ ± 0.16	3.27 ^a^ ± 0.24
10	5.46 ^bc^ ± 0.17	5.22 ^d^ ± 0.05	5.61 ^b^ ± 0.49
12	5.64 ^c^ ± 0.08	5.26 ^d^ ± 0.06	5.57 ^b^ ± 0.07

Data in the same parameter and column with different superscript letters are significantly different (*p* < 0.05).

**Table 3:** Colour values of *C. lentillifera* under 10–30% flower salt concentrations during 12 weeks of storage.

Weeks	Salt Concentration		
	10%	20%	30%
L*			
0	29.92 ^d^ ± 0.34	29.92 ^f^ ± 0.34	29.92 ^d^ ± 0.34
2	24.97 ^b^ ± 0.37	24.91 ^d^ ± 0.30	25.21 ^b^ ± 0.14
4	25.49 ^c^ ± 0.10	25.59 ^e^ ± 0.14	25.72 ^c^ ± 0.11
6	25.57 ^c^ ± 0.06	25.47 ^e^ ± 0.15	25.59 ^c^ ± 0.06
8	21.57 ^a^ ± 0.06	20.44 ^a^ ± 0.09	21.60 ^a^ ± 0.04
10	21.56 ^a^ ± 0.08	20.72 ^b^ ± 0.18	21.61 ^a^ ± 0.05
12	21.48 ^a^ ± 0.06	21.40 ^c^ ± 0.30	21.56 ^a^ ± 0.09
a*			
0	−1.01 ^a^ ± 0.18	−1.01 ^a^ ± 0.18	−1.01 ^a^ ± 0.18
2	0.27 ^b^ ± 0.05	−0.35 ^b^ ± 0.06	−0.55 ^b^ ± 0.06
4	0.36 ^b^ ± 0.05	0.56 ^c^ ± 0.05	0.41 ^c^ ± 0.06
6	0.31 ^b^ ± 0.06	0.53 ^c^ ± 0.08	0.42 ^c^ ± 0.07
8	1.50 ^cd^ ± 0.08	1.42 ^d^ ± 0.06	1.23 ^d^ ± 0.05
10	1.59 ^d^ ± 0.03	1.49 ^de^ ± 0.07	1.30 ^d^ ± 0.09
12	1.45 ^c^ ± 0.09	1.55 ^e^ ± 0.10	1.33 ^d^ ± 0.08
b*			
0	2.87 ^a^ ± 0.79	2.87 ^a^ ± 0.79	2.87 ^a^ ± 0.79
2	3.62 ^b^ ± 0.04	3.88 ^b^ ± 0.11	3.49 ^b^ ± 0.09
4	4.33 ^c^ ± 0.05	3.89 ^b^ ± 0.21	3.57 ^b^ ± 0.08
6	4.46 ^c^ ± 0.13	3.76 ^b^ ± 0.18	3.57 ^b^ ± 0.05
8	6.32 ^d^ ± 0.10	6.40 ^c^ ± 0.10	3.52 ^b^ ± 0.07
10	6.38 ^d^ ± 0.05	6.43 ^c^ ± 0.07	3.55 ^b^ ± 0.11
12	6.40 ^d^ ± 0.05	6.37 ^c^ ± 0.11	4.14 ^c^ ± 0.23

Data in the same parameter and column with different superscript letters are significantly different (*p* < 0.05).

**Table 4:** Hardness and firmness of *C. lentillifera* under 10–30% table salt concentrations during 12 weeks of storage.

Weeks	Salt Concentration		
	10%	20%	30%
Hardness (g force)			
0	1150.71 ^b^ ± 44.36	1150.71 ^b^ ± 44.36	1150.71 ^b^ ± 44.36
2	1240.52 ^c^ ± 47.41	1374.27 ^d^ ± 61.60	1265.11 ^c^ ± 72.33
4	1039.01 a ± 46.85	1056.16 ^a^ ± 69.67	1130.91 ^ab^ ± 96.98
6	1143.69 ^b^ ± 68.83	1228.41 ^c^ ± 79.83	1098.68 ^a^ ± 76.83
8	1074.67 ^a^ ± 78.21	1192.22 ^bc^ ± 70.99	1280.18 ^c^ ± 37.88
10	1265.03 ^c^ ± 87.11	1324.83 ^d^ ± 72.87	1401.26 ^d^ ± 51.88
12	1275.24 ^c^ ± 100.30	1324.45 ^d^ ± 73.45	1411.15 ^d^ ± 38.08
Firmness (g/s)			
0	423.35 ^a^ ± 10.88	423.35 ^a^ ± 10.88	423.35 ^a^ ± 10.88
2	625.88 ^c^ ± 19.63	665.46 ^c^ ± 26.50	624.21 ^b^ ± 24.78
4	525.21 ^b^ ± 22.23	492.63 ^b^ ± 38.61	600.40 ^b^ ± 38.70
6	660.21 ^d^ ± 50.06	758.30 ^e^ ± 46.99	673.29 ^c^ ± 46.99
8	603.74 ^c^ ± 40.32	696.31 ^d^ ± 49.47	731.83 ^d^ ± 41.48
10	724.16 ^e^ ± 22.44	817.98 ^f^ ± 31.83	908.95 ^f^ ± 42.14
12	749.99 ^e^ ± 43.57	816.38 ^f^ ± 45.69	872.88 ^e^ ± 47.80

Data in the same parameter and column with different superscript letters are significantly different (*p* < 0.05).

**Table 5:** Hardness and firmness of *C. lentillifera* under 10–30% sea salt concentrations during 12 weeks of storage.

Weeks	Salt Concentration		
	10%	20%	30%
Hardness (g force)			
0	1150.71 ^de^ ± 44.36	1150.71 ^a^ ± 44.36	1150.71 ^a^ ± 44.36
2	996.70 ^a^ ± 70.46	1174.58 ^a^ ± 79.31	1226.40 ^b^ ± 59.05
4	1126.79 ^bcd^ ± 90.21	1200.75 ^a^ ± 64.44	1341.72 ^c^ ± 49.24
6	1155.77 ^cde^ ± 38.28	1191.21 ^a^ ± 33.84	1337.69 ^c^ ± 48.51
8	1113.60 ^bc^ ± 30.06	1169.82 ^a^ ± 79.12	1322.73 ^c^ ± 42.75
10	1094.19 ^b^ ± 42.87	1213.47 ^a^ ± 83.87	1437.19 ^d^ ± 74.55
12	1197.61 ^e^ ± 68.18	1284.46 ^b^ ± 45.82	1437.79 ^d^ ± 72.56
Firmness (g/s)			
0	423.35 ^a^ ± 10.88	423.35 ^a^ ± 10.88	423.35 ^a^ ± 10.88
2	573.74 ^cd^ ± 40.76	738.54 ^e^ ± 64.72	745.92 ^d^ ± 41.60
4	559.95 ^c^ ± 23.46	577.58 ^b^ ± 39.11	633.01 ^b^ ± 16.33
6	656.40 ^e^ ± 36.17	641.52 ^c^ ± 27.47	767.23 ^d^ ± 26.91
8	587.22 ^d^ ± 16.77	616.38 ^c^ ± 23.39	721.78 ^c^ ± 39.45
10	532.65 ^b^ ± 21.39	639.67 ^c^ ± 34.23	749.26 ^d^ ± 36.08
12	632.97 ^e^ ± 33.70	707.05 ^d^ ± 40.37	836.89 ^e^ ± 31.48

Data in the same parameter and column with different superscript letters are significantly different (*p* < 0.05).

**Table 6:** Hardness and firmness of *C. lentillifera* under 10–30% flower salt concentrations during 12 weeks of storage.

Weeks	Salt Concentration		
	10%	20%	30%
Hardness (g force)			
0	1150.71 ^c^ ± 44.36	1150.71 ^b^ ± 44.36	1150.71 ^a^ ± 44.36
2	1051.52 ^b^ ± 63.62	1214.46 ^c^ ± 64.50	1336.53 ^c^ ± 74.39
4	1075.39 ^b^ ± 62.36	1071.75 ^a^ ± 63.69	1314.33 ^c^ ± 85.23
6	1000.21 ^a^ ± 52.28	1233.60 ^c^ ± 61.72	1220.43 ^b^ ± 28.67
8	1420.99 ^e^ ± 70.43	1525.51 ^e^ ± 51.71	1513.72 ^d^ ± 78.39
10	1244.70 ^d^ ± 65.30	1381.62 ^d^ ± 86.76	1347.16 ^c^ ± 68.99
12	1072.65 ^b^ ± 65.36	1251.98 ^c^ ± 64.38	1330.71 ^c^ ± 93.13
Firmness (g/s)			
0	423.35 ^a^ ± 10.88	423.35 ^a^ ± 10.88	423.35 ^a^ ± 10.88
2	513.18 ^bc^ ± 30.03	574.67 ^c^ ± 18.24	652.26 ^c^ ± 27.06
4	494.32 ^b^ ± 35.14	507.51 ^b^ ± 29.89	611.04 ^b^ ± 33.07
6	499.53 ^b^ ± 29.90	663.45 ^e^ ± 26.22	689.33 ^d^ ± 31.88
8	805.03 ^e^ ± 43.36	809.37 ^g^ ± 33.28	870.71 ^f^ ± 43.63
10	666.96 ^d^ ± 33.75	752.54 ^f^ ± 32.88	724.30 ^e^ ± 34.93
12	526.01 ^c^ ± 26.79	641.61 ^d^ ± 36.15	685.44 ^d^ ± 60.74

Data in the same parameter and column with different superscript letters are significantly different (*p* < 0.05).

**Table 7:** Nutritional analysis of *C. lentillifera* under table salt solution during 12 weeks of storage.

Composition	Fresh	10%			20%			30%		
	*Week 0*	*Week 4*	*Week 8*	*Week 12*	*Week 4*	*Week 8*	*Week 12*	*Week 4*	*Week 8*	*Week 12*
Moisture content (% wet weight)	95.14 ± 0.10	98.45 ± 0.20	98.69 ± 0.01	98.20 ± 0.02	98.24 ± 0.06	98.34 ± 0.35	98.27 ± 0.03	98.08 ± 0.17	98.63 ± 0.05	97.96 ± 0.49
Ash (% DW)	65.64 ± 0.54	21.49 ± 2.22	23.07 ± 0.15	41.78 ± 0.12	27.46 ± 3.53	18.33 ± 1.41	37.88 ± 2.01	31.68 ± 2.19	28.32 ± 4.74	30.09 ± 4.70
Crude fat (% DW)	6.29 ± 0.75	12.38 ± 2.60	15.36 ± 5.04	11.66 ± 4.06	20.38 ± 5.88	18.27 ± 3.03	13.48 ± 3.34	17.04 ± 3.74	13.73 ± 0.83	28.45 ± 3.68
Crude protein (%DW)	4.92 ± 0.49	16.32 ± 0.04	17.59 ± 0.09	12.08 ± 1.12	14.37 ± 1.07	13.01 ± 2.77	18.65 ± 0.19	13.24 ± 0.42	17.36 ± 0.52	11.19 ± 2.21
Crude fibre (%DW)	4.06 ± 0.09	15.72 ± 0.93	17.59 ± 0.56	12.84 ± 1.12	14.70 ± 2.25	14.84 ± 3.03	15.46 ± 0.05	14.68 ± 2.92	16.34 ± 2.35	12.36 ± 2.36
Carbohydrate (% DW)	19.09 ± 0.78	34.09 ± 3.85	26.39 ± 5.65	21.64 ± 3.17	23.09 ± 15.67	35.56 ± 1.24	14.52 ± 1.43	23.36 ± 18.42	24.26 ± 5.74	17.91 ± 3.80

**Table 8:** Microbiological analysis of *C. lentillifera* under three salt types and salt concentrations during 12 weeks of storage.

Weeks	Table Salt Concentration			Sea Salt Concentration			Flower Salt Concentration		
	10%	20%	30%	10%	20%	30%	10%	20%	30%
Aerobic plate count, (CFU/g)									
0	1.90 × 10^2^	2.08 × 10^2^	2.43 × 10^2^	2.90 × 10^2^	3.15 × 10^2^	3.15 × 10^2^	1.74 × 10^2^	1.54 × 10^2^	3.70 × 10^2^
4	<10	<10	<10	1.74 × 10^2^	1.48 × 10^2^	1.00 × 10^2^	<10	<10	<10
12	3.26 × 10^2^	2.08 × 10^2^	<10	3.26 × 10^2^	1.54 × 10^2^	<10	3.26 × 10^2^	1.98 × 10^2^	<10
Yeast, (CFU/g)									
0	<10	<10	<10	<10	<10	<10	<10	<10	<10
4	<10	<10	<10	<10	<10	<10	<10	<10	<10
12	<10	<10	<10	<10	<10	<10	<10	<10	<10
Mould, (CFU/g)									
0	1.70 × 10^2^	1.23 × 10^2^	<10	1.70 × 10^2^	2.04 × 10^2^	1.60 × 10^2^	<10	<10	<10
4	<10	<10	<10	<10	<10	<10	<10	1.63 × 10^2^	1.95 × 10^2^
12	<10	<10	<10	<10	<10	<10	2.00 × 10^2^	2.40 × 10^2^	2.46 × 10^2^
Coliform bacteria, (MPN/g)									
0	<3	<3	<3	<3	<3	<3	<3	<3	<3
4	<3	<3	<3	<3	<3	<3	<3	<3	<3
12	<3	<3	<3	<3	<3	<3	<3	<3	<3

**Table 9:** Elemental analysis of three kinds of salt via SEM-EDS.

Elements	Sample Type	Weight (%)	Atomic (%)
Oxygen (O)	Table salt	2.02	3.73
	Sea salt	14.41	24.20
	Flower salt	8.61	15.12
Sodium (Na)	Table salt	32.58	41.83
	Sea salt	25.14	29.38
	Flower salt	27.93	34.13
Chlorine (Cl)	Table salt	65.40	54.44
	Sea salt	57.09	43.27
	Flower salt	61.17	48.46
Magnesium (Mg)	Table salt	-	-
	Sea salt	1.70	1.88
	Flower salt	1.29	1.49
Sulphur (S)	Table salt	-	-
	Sea salt	0.88	0.74
	Flower salt	0.54	0.47
Potassium (K)	Table salt	-	-
	Sea salt	0.46	0.32
	Flower salt	0.46	0.33
Calcium (Ca)	Table salt	-	-
	Sea salt	0.31	0.21
	Flower salt	-	-

## Data Availability

Not applicable.
